# National Survey of the Smoking Cessation Services in Italy

**DOI:** 10.3390/ijerph6030915

**Published:** 2009-02-26

**Authors:** Alessandra Di Pucchio, Enrica Pizzi, Giordano Carosi, Monica Mazzola, Donatella Mattioli, Roberta Pacifici, Simona Pichini

**Affiliations:** Therapeutic Research and Medicines Evaluation Department, Italian Epidemiological Observatory on Tobacco, Alcohol and Drugs of abuse, Istituto Superiore di Sanità, Viale Regina Elena 299, 00161 Rome, Italy; E-Mails: enrica.pizzi@iss.it (E.P.); giordano.carosi@iss.it (G.C.); monica.mazzola@iss.it (M.M.); donatella.mattioli@iss.it (D.M.); simona.pichini@iss.it (S.P.); roberta.pacifici@iss.it (R.P.)

**Keywords:** Smoking cessation services, tobacco smoking, tobacco use cessation

## Abstract

This investigation is aimed at providing information about structural and organizational characteristics of smoking cessation services (SCS) set up within the Italian National Health Service. Local health units and hospitals are the main institutions connected with SCS which are mainly located within the Department of Drug Addiction and the Department of Lung and Breath Care. SCS provide different tobacco-use cessation programs. Although pharmacotherapy is always used, a combination of therapeutic treatments is highly preferred. This study shows the importance of maintaining a national coordination among different SCS supporting their activity and encouraging the start up of additional services throughout the country.

## 1. Introduction

In 2007, about 22% of adults aged 15 and older were active smokers in Italy [1–3]. Although smoking prevalence has dropped significantly since the 1950s, the decline has been much slower in the last decade [4,5]. In 2007, more than 560,000 smokers quitted smoking, leading to an increase in the percentage of ex-smokers from 17.5% to 18.4% in 2008 [1–3]. Despite this, smoking remains a public health problem in Italy.

It has been demonstrated that Smoking Cessation Services (SCS) play a key role in the reduction of smoking habit [6–9]. International studies indicate that in the activity of SCS many factors are significant determinants of “good practice” and positive outcomes: the stage of service development and the form of intervention offered by the services are significant factors influencing both reach and cessation rate [8,10].

A survey carried out in 143 Italian SCS showed that all therapeutic approaches offered by SCS are helpful in smoking cessation habits, in particular pharmacological therapies associated with group therapies [8].

In Italy, several cessation programs and services are currently available [11]. A 2008 DOXA (Italian Institute of Statistical Research and Public Opinion Analysis) survey showed that 80.6% of people think that SCS access is an impressive prevention measure initiated by the Government to both reduce smoking and to help people to quit smoking [2].

However, there are differences in SCS activities since no national comprehensive tobacco control program has been fully implemented. Indeed, several SCS are not adequately funded and their activities are only periodically implemented. Therefore, it is imperative to help physicians and health professionals in their effort of informing and helping smokers to quit smoking [12,13].

Since 2000, the Italian Epidemiological Observatory on Tobacco, Alcohol and Drugs of Abuse of Istituto Superiore di Sanità (OssFAD, ISS) is promoting a nation-based survey to list and update available SCS and to investigate their characteristics in evaluating the effectiveness of smoking cessation programs offered[8,11,14–17].

To support health professionals, the OssFAD updated guidelines on smoking cessation activities published for the first time in 2002 [18]. The guidelines stated that general practitioners should regularly monitor smoking status of known smokers, advise them to stop at every opportunity, arrange follow-ups for those intending to make an attempt to quit, and recommend use of pharmacotherapy. They should also provide assistance in the form of referral to a specialist clinic for those smokers who wish to stop [12].

Since 1998–2000, the struggle against tobacco has been an important objective of the Italian National Health Plan. However, differently from other countries no national treatment system is yet available [3,18,19]. Consequently, the number of nationwide SCS and their treatment programs are not fully established at a national level and there are differences in the cost and reimbursement of treatments. Indeed, while the reimburse of individual counseling and group therapy can be partial or complete depending on SCS, no refund is provided for smoking cessation medication.

This investigation was aimed at providing information about structural and organizational characteristics of smoking cessation services set up within the Italian National Health Care Service (Servizio Sanitario Nazionale -SSN).

## 2. Experimental Section Methods

### 2.1. Study Participants

To obtain structural and organizational information about SCS and monitor their activities, telephone interviews were held with principal coordinators of each Italian SCS. To contact all the coordinators we used an available and updated list of Italian SCS (including the name of principal coordinators) [11]. A total of 267 SCS were counted in a census. All the SCS were contacted by phone and all the centres responded to our survey. Interviews were held by ad hoc trained specialists between January and April 2008.

### 2.2. Data Collection

Data were collected using a structured interview form (the translated full questionnaire is given in Appendix 1) including the following information:

#### 2.2.1. SCS Locations and Additional Information on Service’s Organization

This section asked for information about: site of the SCS (local health unit, hospitals and other health centres); general information on location (including address, telephone number, email address); service’s name, principal coordinator’s name; service activation year; legal authorization that regulates their activities.

#### 2.2.2. Service’s Access Modalities

This section sought information about: registration procedures and consulting hours; costs to access the tobacco-use cessation program.

#### 2.2.3. Smoking Cessation Programs Offered

This section collected data on the range of smoking cessation interventions delivered by the service: pharmacotherapies, individual counseling, group therapy and other treatments such as acupuncture, relaxation therapy and hypnotherapy. Additionally included questions were on frequency and duration of therapeutic treatment delivered including number, frequency and duration of therapy sessions.

#### 2.2.4. SCS Staff and Professional’s Qualification of Specialists

This section collected information about staff working for the SCS such as number of specialists working in SCS and their professional qualification (physician, clinical psychologists, other professionals – e.g. administrative, trained nurse, nurse’s aide, physiotherapist, sociologist, trained educator, other).

#### 2.2.5. Number of Smokers Treated in 2007

Respondents were asked to state numbers of individuals who had received at least one treatment to quit smoking in 2007 at the specific SCS site.

### 2.3. Data Analysis

All data were analyzed by SPSS 15.0 software and descriptive analyses of principal collected data were carried out.

## 3. Results

### 3.1. SCS Locations and Organization

In April 2008, all 267 SCS nationwide were contacted by phone and all of them responded to our survey. Table 1 shows SCS locations and other information on the service’s organization. As shown in Table 1, local health units and hospitals were the main institutions connected to SCS. Specifically, SCS were mainly located within the Department of Drug Addiction and the Department of Lung and Breath Care (Table 1).

**Figure 1.**
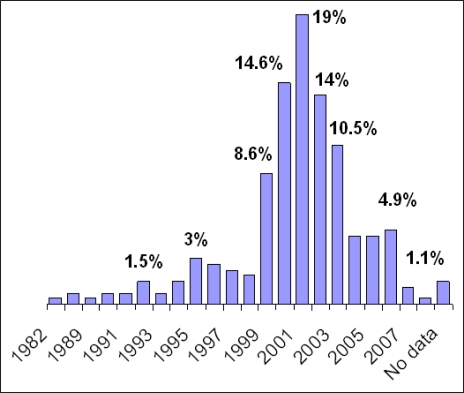
SCS Activation Period.

The first SCS begun to operate at the end of the 90s and their number increased between 1999 and 2003 (Figure 1). Furthermore, a majority of SCS (67.8%) started up their activity either under specific Regional and Health Agency Action regulations or as “mission” of their Departments (e.g. Department of Drug Addiction, Prevention and Health Care Department, etc.). In contrast, 10.5% of SCS were activated without any specific starting-up regulations, while for 21.9% of SCS data were not available. A further analysis showed that SCS operate under a variety of names: “Anti-Smoking Centre/Centre for Smoking Cessation” (52.8%), “Clinic for Tobacco Addiction/Clinic for Smoking Cessation (24%) and additional names for the remaining 23.2%.

### 3.2. Service Access Modalities

The modalities utilized to contact and to have access to the SCS are shown in Table 2. To obtain information or to schedule appointments, individuals had the possibility to contact the SCS by telephone (33.9%), going directly to the SCS (1.3%) or to choose between the two mentioned options (64.8%).

In order to access the tobacco-use cessation programs, 49.8% of SCS asked for a requisition from a family doctor, 44.6% of SCS had free admission while 4.8% of SCS practiced a combination of the above. In addition, the analysis showed that on average, SCS work four days a week; 36.3% SCS are available three days a week, 43.1% four-five days a week and 3.4% six-seven days a week. Regarding costs of accessing the tobacco-use cessation programs, 59% of the SCS required patient's contribution (e.g., ticket, association fee), 22% was cost-free (at public assistance’s expenditure) and 19% a combination of the two (Table 2). These differences were dependent on SCS location and on specific regional health plan action regulations.

### 3.3. Smoking Cessation Programs

**Figure 2.**
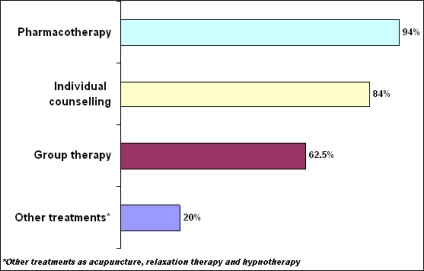
Smoking Cessation Treatments.

Figure 2 shows the prevalence of different smoking cessation treatments used by SCS. Interestingly, pharmacotherapies proved to be the most utilized therapies (94%), followed by individual counseling (84%) and then group therapy (64%).

Of note, although treatments always included pharmacotherapies, a combination of therapeutic treatments was highly preferred (97%) (Table 3).

### 3.4. SCS Staff and Professional’s Qualification

**Figure 3.**
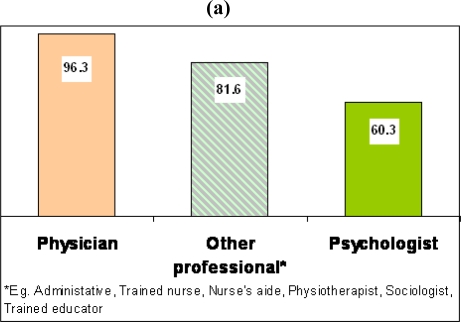

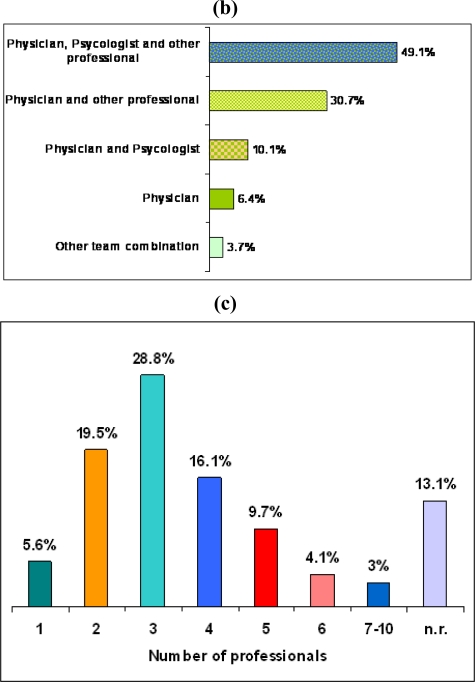
Professionals’ Qualification and Team Compositions.

The breakdown of the staff and their professional qualification working at the SCS is shown in Figure 3. The highest percentage of staff were physicians (96.3%) followed by other professionals (81.6%) such as administrative personnel, nurse, nurse assistant, physiotherapist, sociologist, trained educator and clinical psychologists (60.3%) (Figure 3a). It is worth mentioning that majority of SCS (96.6%) were led by at least one physician who operated in a team with clinical psychologists (59.2%) and other professionals (30.7%) and only in 6.4% of SCS the physicians were working by themselves (Figure 3b). In fact, an average of three different specialists was the most representative combination of SCS staff (Figure 3c).

### 3.5. Number of Individuals Treated for Smoking Habit during 2007

In 2007, over 15,000 individuals requested help from SCS. The analysis showed that 47.7% of SCS treated 10–50 smokers, 28.9% between 50–100 smokers and 23.4% more than 100 smokers. Overall, each SCS treated an average of seventy smokers, who received at least one treatment to quit smoking.

## 4. Discussion and Conclusions

This study investigated structural and organizational characteristics of smoking cessation services (SCS) set up within the Italian National Health Service and provided their characteristics – information that was previously not available.

Findings from our study suggest that there are differences in SCS activities as no national comprehensive tobacco control programs have been fully implemented. Indeed, several SCS are not adequately funded and their activities are only periodically implemented. Our findings are supported by a recent study [19] which reports that although Italy has an official written policy on tobacco dependence treatment, no national treatment system is still available.

The first SCS begun to operate at the end of the 90s and their number increased considerably between 1999 and 2003. However, although new SCS are constantly activated, others are being closed down, resulting in an overall decrease in the number of SCS in the last three years. Of note, a majority of SCS (67.8%) started up their activity either under specific Regional and Health Agency Action regulations or as “mission” of their Departments (e.g. Department of Drug Addiction, Prevention and Health Care Department, etc.).

SCS deliver a range of smoking cessation treatments as pharmacotherapy, individual counseling and group therapy. Although there is no evidence of efficacy of acupuncture and hypnosis techniques in smoke cessation habits, 17.6% SCS uses these techniques in combination with other treatments, particularly in those patients who cannot use medications.

Our analysis underlines that due to differences in SCS locations and specific regional health plan action regulations, the costs of access tobacco-use cessation programs differ from an SCS to another. Although 22% of SCS delivers cost-free treatments, smoking cessation treatment can be very expensive reducing smoker treatment accessibility.

In 2007, over 15,000 individuals received at least one treatment within SCS to quit smoking. This number could increase through a more extensive communication campaign with the general practitioners. In fact, guidelines on smoking cessation activities stated that general practitioners represent the cornerstone of the SCS. They are essential in prompting quitting attempts by delivering brief opportunistic advice to their smoking patients and are crucial in directing these smokers to their local specialist service[12–13,18].

In the last years, SCS have been well established and have developed a wide range of treatment models. Although the organizational process of SCS has improved access to the services, there are still concerns about quality standards and comparative performance across the services’ network.

Our survey suggests that since SCS were launched, there have been significant developments and SCS have an important role on smoking cessation rates at population level. However, various institutional policies would facilitate service development and improvement. First, it could be fundamental to develop a specialized treatment system delivered by trained professionals covering the whole country. Therefore, SCS should have a dedicated staff to provide tobacco dependence treatments. Finally, total reimbursement of medications could facilitate and increase the number of smokers who try to quit. Taken together, adequate training, resources and feedback to ensure that providers consistently deliver effective treatments could improve and develop SCS services.

These results are important to initiate a comparison of activities and characteristics of SCS at both national and international level. In addition, our research is helpful for starting a monitoring activity on qualities and efficacy of therapeutic treatments provided by different SCS. Finally, this study shows the importance of a national coordination among the network of SCS supporting their activity and encouraging activation of additional services throughout the country. Present data emphasize the need for constant monitoring of SCS activities and their effort to increase the access to local services.

Future studies will not only reveal more about SCS activities but will also help in indicating significant determinants of “good practice” and positive outcomes.

## Figures and Tables

**Table 1.** SCS Characteristics.

Geographic availability of SCS	%
North	59
Central	22
South and Island	19
**Institution connected to SCS**	
Local health units	55.8
Hospital	40.8
Other*	3.4
**SCS location**	
Department of Drug Addiction	29.9
Department of Lung and Breath Care	29.6
Other locations**	40.5

* Co-operation between hospital and Local Health Unit

** E.g. Department of Cardiology, Oncology, co-operation between additional services, etc.

**Table 2.** Service Access Modalities.

SCS Characteristics	%
**Registration procedures**	
By telephone	33.9
Direct to SCS	1.3
A combination of both	64.8
**Registration modalities**	
Written family doctor request	49.8
Free admission	44.6
A combination of both	4.8
Data not available	0.8
**Consulting days per week**	
1–3 days	36.3
4–5 days	43.1
6–7 days	3.4
Data not available	17.2
**Costs of access to smoking cessation programs**	
Patient’s contribution required	59
Cost-free	22
A combination of both	19

**Table 3.** Tobacco-use Cessation Programs.

Combinations of therapeutic treatment	%
Pharmacotherapy + Individual counseling +group therapy	32.2
Pharmacotherapy + Individual counseling	22.1
Pharmacotherapy + Individual counseling +group therapy + other treatment*	17.6
Other combinations	28.1

* Other treatments refer to acupuncture, relaxation therapy and hypnotherapy

## Appendix 1. Structured interview form

**Table:**

**SCS location/setting and information on service organization**
Institution connected to SCS (Local health units/hospital/other):
Department:
SCS denomination and Address:
Principal Coordinator (Surname/ First name/ Address):
Service activation year: ________
Legal authorization that regulates SCS activities:
**Service access modalities**
Registration procedures: □ By telephone □ Direct to SCS □ Other
Registration modalities: □ Written family doctor request □ Free admission □ Other__________
Consulting days per week: □ 1–3 days □ 4–5 days □ 6–7 days □ other____________/___________________
Costs to access the tobacco-use cessation program: □ Patient’s contribution required □ Cost-free □ Other________
**Smoking cessation programs**
□ Pharmacotherapy__________________________
□ Individual counseling__________________________
□ Group therapy__________________________
□ Other treatments (specify) __________________________
*For all programs specify frequency and duration of therapeutic treatment*
**SCS Staff and Professional’s Qualification**
Number and Professional’s qualifications:
□ N. __ Physician
□ N. __ Clinical Psychologist
□ N. __ Other professional (specify) __________________________
□ N. __ Other professional (specify) __________________________
**Number of Smokers treated in 2007**
Numbers of individuals who received at least one treatment to quit smoking in 2007: ______
